# Investigation into Bioactive Selenium Species and the Mechanism of Action Behind Selenium-Enriched Rapeseed Flowering Stalks Alleviating Cadmium-Induced Toxicity in Mouse Sertoli Cells

**DOI:** 10.3390/antiox14111297

**Published:** 2025-10-28

**Authors:** Huatao Che, Yiqing Lu, Tong Li, Xiaoli Fang, Xinfa Wang, Hanzhong Wang, Xiaoling Dun, Zhenna Chen

**Affiliations:** 1 Key Laboratory of Biology and Genetic Improvement of Oil Crops, Oil Crops Research Institute of the Chinese Academy of Agricultural Sciences, Ministry of Agriculture and Rural Affairs, Wuhan 430062, China; 82101225133@caas.cn (H.C.);; 2 Institute of Crop Science, Zhejiang University, Hangzhou 310058, China; 3 Hubei Hongshan Laboratory, Wuhan 430070, China

**Keywords:** selenium-enriched rapeseed, cadmium, organic selenium, mouse Sertoli cells, RNA-Seq, mechanism

## Abstract

Cadmium (Cd) is a recognized environmental contaminant, present in soil, water, and food, which has been reported to cause male reproductive damage in vivo and vitro. Selenium-enriched rapeseed flowering stalks exhibit protective effects against Cd-induced reproductive damage, yet the bioactive components and underlying mechanisms remain unclear. We optimized the process of obtaining the crude extract (CE) via single-factor experiments. Subsequent bioassay-guided fractionation identified the water extract (WE) as significantly more effective in alleviating Cd-induced cytotoxicity compared to the petroleum ether extract, ethyl acetate extract, and n-butanol extract. High-performance liquid chromatography–inductively coupled plasma mass spectrometry (HPLC-ICP-MS) analysis revealed that WE contained the highest contents of methylselenocysteine (MeSeCys) and selenocystine (SeCys_2_) among four fractions. Both MeSeCys and SeCys_2_ exhibited protective effects against Cd-induced cytotoxicity. To further elucidate the underlying mechanisms, network pharmacology, RNA-Seq, qPCR, and Western blotting analysis were employed. The results revealed that WE exhibited good free radical scavenging capabilities, and the protective mechanisms of WE, MeSeCys, and SeCys_2_ against Cd-induced cytotoxicity were related to a reduction in oxidative damage, the inhibition of the ERK/p38 MAPK signaling pathway, and the suppression of cell cycle arrest, inflammation, and apoptosis triggered by Cd exposure. Collectively, these findings suggest that selenium-enriched rapeseed flowering stalks may serve as a promising dietary supplement in the prevention of Cd-induced reproductive toxicity.

## 1. Introduction

Cadmium (Cd) is a widespread metal pollutant that poses a severe threat to human health [1]. Studies have shown that Cd accumulates in the human body through foods, water, air, and occupational exposure [2], and Cd exposure can damage various organs, including the liver, kidney, and testes, due to its long half-life [3]. Numerous studies have demonstrated that Cd exposure can cause serious toxicity to the male reproductive system, including reduced testicular weight, disruptions to spermatogonia, and impaired sperm production [4]. The cytotoxic mechanisms underlying Cd-induced damage to the reproductive system primarily encompass oxidative stress, DNA damage, damage to the mitogen-activated protein kinase (MAPK) pathway, inflammation, and apoptosis [5,6].

Selenium, an essential element in human survival, plays important roles in maintaining male reproductive health [7], and selenium supplementation can alleviate the toxicity of Cd in the testes [8]. For instance, sodium selenite has been found to alleviate testicular toxicity caused by Cd exposure through decreasing oxidative stress levels [9]. Additionally, various selenium-enriched foods, such as probiotics [10], rice [11], and seaweed [12], have been rapidly developed for the market to meet human nutritional needs; these are able to alleviate Cd damage in organisms. For instance, a selenium-enriched plant (*Cardamine hupingshanensis*) was shown to significantly alleviate Cd-induced liver injury in mice by regulating SLC7A11/GPX4 signaling and lipid peroxidation [13]. It is important to emphasize that the biological activity of selenium in organisms depends on its chemical form, with organic selenium species exhibiting higher bioavailability and biological activity compared to inorganic selenium. Currently, researchers mainly focus on inorganic selenium, and the efficacy and underlying mechanisms of organic selenium species, particularly those derived from natural plant sources, remain poorly understood. Therefore, there is an increasing focus on the use of high-quality selenium-enriched food in alleviating Cd-induced toxicity in the male reproductive system.

For years, *Brassicaceae* plants have represented a key research subject due to their nutritional value and pharmacological effects [14]. Rapeseed (*Brassica napus* L.) belongs to the *Brassicaceae* family and is currently acknowledged as the third-largest oil crop worldwide, with an annual yield of approximately 75 million tons [15]. Relevant research has demonstrated that rapeseed is enriched in multiple nutrients, such as vitamin E, minerals, fatty acids, amino acids, and glucosinolates [16]. It exhibits diverse physiological properties such as antioxidant [17], antimicrobial [14], and anticancer activities [18]. Studies have shown that rapeseed has a unique capability for selenium enrichment [19]. Zhan et al. identified the selenium species present in selenium-enriched rapeseed flowering stalks as comprising five different selenium compounds, including MeSeCys, selenomethionine (SeMet), SeCys_2_, selenite, and selenate [20]. Up until now, the impact of selenium-enriched rapeseed on Cd-induced damage to the reproductive system and the underlying mechanisms have not been studied.

Within the seminiferous epithelium, Sertoli cells represent the sole somatic cell population, playing pivotal roles in providing structural support and generating energy substrates during spermatogenesis [21]. These cells are widely utilized as in vitro models for investigating the reproductive toxicity of environmental pollutants [22] and hazardous metals [23]. In the present study, we explored the positive effects of selenium-enriched rapeseed flowering stalk extract and its main organic selenium species with regard to reducing Cd-induced damage in mouse Sertoli cells (TM4 cells). We optimized the process of extracting bioactive components from rapeseed flowering stalks, and the most active fraction was identified based on a bioassay-guided fractionation method. Under the optimal extraction parameters, the selenium species present in the most active fraction was further characterized via (HPLC-)ICP-MS. Subsequently, transcriptomics and cell assays were employed to elucidate the mechanisms underlying selenium-enriched rapeseed flowering stalks and organic selenium species reducing Cd-induced toxicity in TM4 cells. Our findings offer novel chemical and biological insights into the positive effects of selenium-enriched rapeseed flowering stalks in preventing Cd-induced damage to male infertility, aiding in the cultivation and healthy development of rapeseed.

## 2. Materials and Methods

### 2.1. Chemicals and Reagents

Cadmium chloride (CdCl_2_) was obtained from Aladdin Reagent Co., Ltd. (Shanghai, China). The cell counting kit-8 (CCK-8) and the CyQUANT™ NF cell proliferation assay kit were purchased from Biosharp Biotechnology Company (Beijing, China). Nitric acid, methanol, petroleum ether, ethyl acetate, and n-butanol were provided by China National Pharmaceutical Group Chemical Reagent Co., Ltd. (Beijing, China). The Annexin V-FITC/PI apoptosis kit and cell cycle assay kit were purchased from Elabscience Biotechnology Co., Ltd. (Wuhan, China). 2,2-diphenyl-1-picrylhydrazyl (DPPH), 2,2′-azino-bis-3ethylbenzthiazoline-6-sulphonate (ABTS), reactive oxygen species (ROS), malonic dialdehyde (MDA), and L-lactate dehydrogenase (L-LDH) were provided by Beijing Solarbio Science and Technology Co., Ltd. (Beijing, China).

### 2.2. Optimization of Extraction Conditions

Selenium-enriched rapeseed flowering stalks were obtained from the Oil Crops Research Institute, Chinese Academy of Agricultural Sciences. The harvested flowering stalks were ground into a powder, which was freeze-dried using a freeze-dryer and subsequently kept refrigerated at 4 °C for subsequent experiments. The crude extract (CE) was prepared via ultrasound-assisted extraction employing methanol as the solvent. Following extraction, methanol was entirely evaporated and removed using a rotary evaporator to obtain a dry residue. Then, the dried extract was reconstituted in DMEM/F-12 medium and further diluted with DMEM/F-12 medium to achieve the required working concentrations. Single-factor experiments were conducted to study the effect of the methanol concentration (30–90%), solid–liquid ratio (1:10–1:25 (*w*/*v*) (g/mL)) and extraction time (6–18 min) on the efficacy of the obtained CE in mitigating CdCl_2_-induced cytotoxicity, with DMEM/F-12 medium serving as the control.

### 2.3. Bioassay-Guided Fractionation

Based on the results of the single-factor experiments reported above, the CE was prepared under the optimal extraction conditions (methanol concentration: 50%; solid–liquid ratio: 1:15; extraction time: 9 min). Following extraction, methanol was entirely evaporated and removed using a rotary evaporator to obtain the CE powder. Then, 2 g of CE powder and 30 mL of distilled water were added into a 50 mL PTFE vessel, and the CE was sequentially fractionated using the liquid–liquid extraction method. Four fractions were obtained: petroleum ether extract (PEE), ethyl acetate extract (EAE), n-butanol extract (BE), and WE. Then, the viability of TM4 cells treated with each fraction was, respectively, detected via a CCK-8 assay and DNA assay. Concurrently, according to the (HPLC-)ICP-MS analysis method [20], the total selenium content was detected using ICP-MS, and different selenium species were detected in different fractions via HPLC-ICP-MS. Please refer to the Supplementary Materials for more detail.

### 2.4. Antioxidant Activity Assay

The DPPH or ABTS assay was performed according to the instructions in the reagent kit. Firstly, 10 μL WE solution was mixed with 190 μL DPPH or ABTS solution. Then, the mixture was treated and detected at 515 nm or 405 nm using a microplate reader (Thermo Scientific Fisher, Waltham, MA, USA).

### 2.5. Cell Assays

#### 2.5.1. CCK-8 Assay and DNA Assay

TM4 cell culture medium was purchased from Procell Life Science & Technology Co., Ltd. (Wuhan, China), and detailed information regarding cell culture and treatment is given in the Supplementary Materials.

#### 2.5.2. Flow Cytometry Analysis

After being exposed to various treatments for 24 h, TM4 cells were collected and cleaned with PBS three times. After centrifugation (2990 rpm × 5 min), the cells were treated using the cell apoptosis assay kit and cell cycle assay kit and detected via flow cytometry (Beckman Coulter, Brea, CA, USA). Details are provided in the Supplementary Materials.

#### 2.5.3. Detection of ROS, MDA, and L-LDH

After being exposed to various treatments for 24 h, TM4 cells were collected and washed with PBS three times. The intracellular ROS level was determined using an ROS assay kit and then detected using an automatic microplate reader (Biotek SynergyH1, Winooski, VT, USA). For MDA and L-LDH detection, the cells were treated using the MDA assay kit and L-LDH assay kit, respectively. Next, the detection of MDA was carried out by measuring the absorbance at 532 nm and 600 nm, while the LDH levels were detected as the absorbance at 450 nm, using a microplate reader (Scientific Multiskan Sky, Thermo, Waltham, MA, USA).

### 2.6. RNA-Seq Analysis

TM4 cells subjected to different incubation conditions were collected for RNA-seq analysis using the MGISEQ-2000 platform (MGI Tech Co., Ltd., Shenzhen, China). To ensure the reliability of the results, three replications were performed. Differential expression analysis was performed using DESeq2 (v1.4.5) with *p* ≤ 0.05.

### 2.7. qRT-PCR Analysis

Total RNA was extracted from TM4 cells using the Fastpure^®^ cell/Tissue total RNA isolation kit V2 (Nanjing, China). Then, reverse transcription was performed, employing the PrimeScript RT reagent kit with a gDNA eraser (Nanjing, China). qRT-PCR was quantified using the taq pro universal SYBR qPCR master mix kit (Nanjing, China) and analyzed using the Roche LightCycler96 instrument (Roche, Basel, Switzerland). The primer sequences are detailed in Table S1.

### 2.8. Western Blot Analysis

The protein levels of ERK, p38, JUN, and P27 in TM4 cells were analyzed via a Western blot assay. Glyceraldehyde-3-phosphate dehydrogenase (GAPDH) was used as the internal reference. The procedure is described in the Supplementary Materials.

### 2.9. Statistical Analysis

All tests were performed in triplicates, and all data are presented as the mean ± standard deviation. Statistical analysis was performed via one-way analysis of variance (ANOVA) followed by Dunnett’s multiple comparison test. Data were analyzed using GraphPad Prism 9.0 (GraphPad Software, San Diego, CA, USA). *p* < 0.05, *p* < 0.01, and *p* < 0.001 were selected for statistical significance.

## 3. Results

### 3.1. Optimization of Extract Conditions

TM4 cells were used as the cell model, and the extraction conditions were optimized via a CCK-8 assay and DNA assay. Firstly, we explored the influence of CdCl_2_ concentration on cell viability. The results revealed that cell viability decreased along with the increase in CdCl_2_ concentration, and the half-maximal inhibitory concentration (IC50) of CdCl_2_ was 6.96 μmol/L (Figure S1). Therefore, 7 μmol/L CdCl_2_ was selected for subsequent experiments.

**Figure 1:**
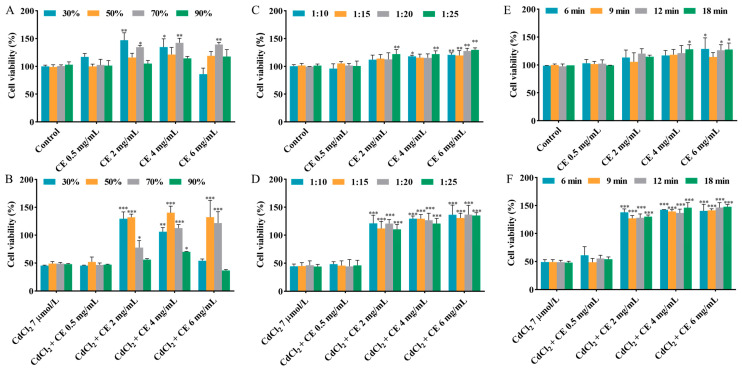
Effects of different extraction conditions on the viability of TM4 cells. The effects of methanol concentration on the viability of cells treated with CE alone (**A**) and co-treated with CE and CdCl_2_ (**B**). The effects of the solid–liquid ratio on the viability of cells treated with CE alone (**C**) and co-treated with CE and CdCl_2_ (**D**). The effects of the extraction time on the viability of cells treated with CE alone (**E**) and co-treated with CE and CdCl_2_ (**F**). The results are expressed as means ± SD (*n* = 3). For (**A**,**C**,**E**), * *p* < 0.05, ** *p* < 0.01, and *** *p* < 0.001 vs. control group. For (**B**,**D**,**F**), * *p* < 0.05, ** *p* < 0.01, and *** *p* < 0.001 vs. CdCl_2_ group.

Methanol has been widely utilized as a solvent in the extraction of active compounds from raw materials [24]. The extraction efficiency can be influenced by several factors, including the solvent concentration, solid–liquid ratio, and extraction time [25]. The influence of methanol concentration on cell viability in the individual exposure group and co-exposure group was studied, with the methanol concentration ranging from 30% to 90%. According to Figure 1A,B and Figure S2A,B, compared to the CdCl_2_ group, co-exposure with 2–6 mg/mL CE markedly increased the cell viability when the methanol concentration was 50–70%. Furthermore, when the methanol concentration was fixed at 50%, the obtained CE did not influence the cell viability. Therefore, 50% methanol was selected.

As shown in Figure 1C,D and Figure S2C,D, the influence of the solid–liquid ratio (1:10–1:25) on the cell viability was examined in the individual exposure group and co-exposure group. It is worth noting that 2–6 mg/mL CE significantly mitigated the damage caused by CdCl_2_ to cell viability across the entire solid–liquid ratio range studied. Considering cost and cell viability, the optimal solid–liquid ratio was selected as 1:15.

The impact of the extraction time, varying within the range of 6–18 min, on cell viability in the individual exposure group and co-exposure group was also studied. According to Figure 1E,F and Figure S2E,F, when the CE concentration was in the range of 2–6 mg/mL, the CE significantly reduced the damage caused by CdCl_2_ to cell viability at different extraction times. Additionally, when the extraction time was fixed at 9 min, it can be seen that the CE did not influence the cell viability. Taking into account cell viability and the high extraction throughput, 9 min was chosen as the extraction time.

### 3.2. Bioassay-Guided Fractionation

**Figure 2:**
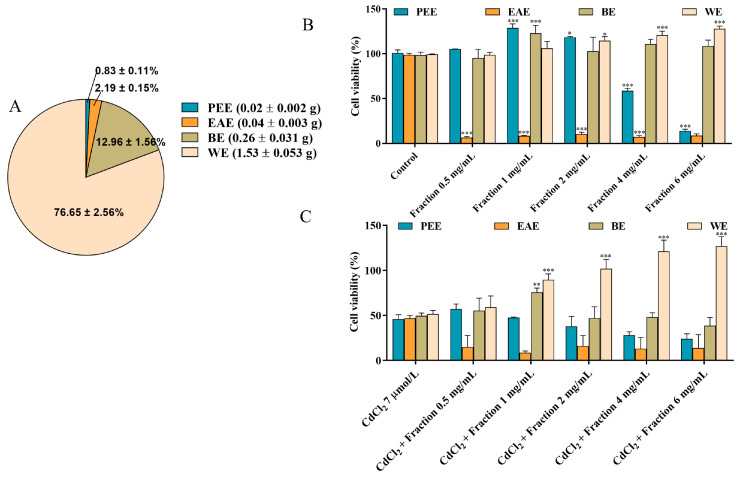
Bioassay-guided fractionation. (**A**) The yields of PEE, EAE, BE, and WE. (**B**) The effects of PEE, EAE, BE, and WE on the cell viability. (**C**) The effects of PEE, EAE, BE, and WE on Cd-induced cytotoxicity in TM4 cells. The results are expressed as means ± SD (*n* = 3). For (**B**), * *p* < 0.05, and *** *p* < 0.001 vs. control group. For (**C**), ** *p* < 0.01, and *** *p* < 0.001 vs. CdCl_2_ group.

The composition and contents of substances in different fractions are closely related to the solvent polarity and the solubility of components in the respective solvent [26]. In this study, the CEs obtained under the optimal extraction conditions were further fractionated using different solvents, resulting in the acquisition of PEE, EAE, BE, and WE. According to Figure 2A, the yields of PEE, EAE, BE and WE were determined to be 0.83 ± 0.11%, 2.19 ± 0.15%, 12.96 ± 1.56%, and 76.65 ± 2.56%, respectively, with WE exhibiting the highest yield among the fractions. To identify the most effective activity fraction, we used a CCK-8 assay and DNA assay to assess and compare the alleviating influence of each fraction toward CdCl_2_-induced reduction in cell viability. As shown in Figure 2B,C and Figure S3, WE intervention notably increased cell viability, indicating that WE significantly protected the cells against CdCl_2_-induced toxicity. Additionally, when the incubation concentration of WE ≤ 1 mg/mL, WE did not influence cell viability. These findings suggest that the active components responsible for alleviating CdCl_2_-induced cytotoxicity were predominantly present in the WE fraction.

### 3.3. Selenium-Containing Bioactive Components in the Extract

**Figure 3:**
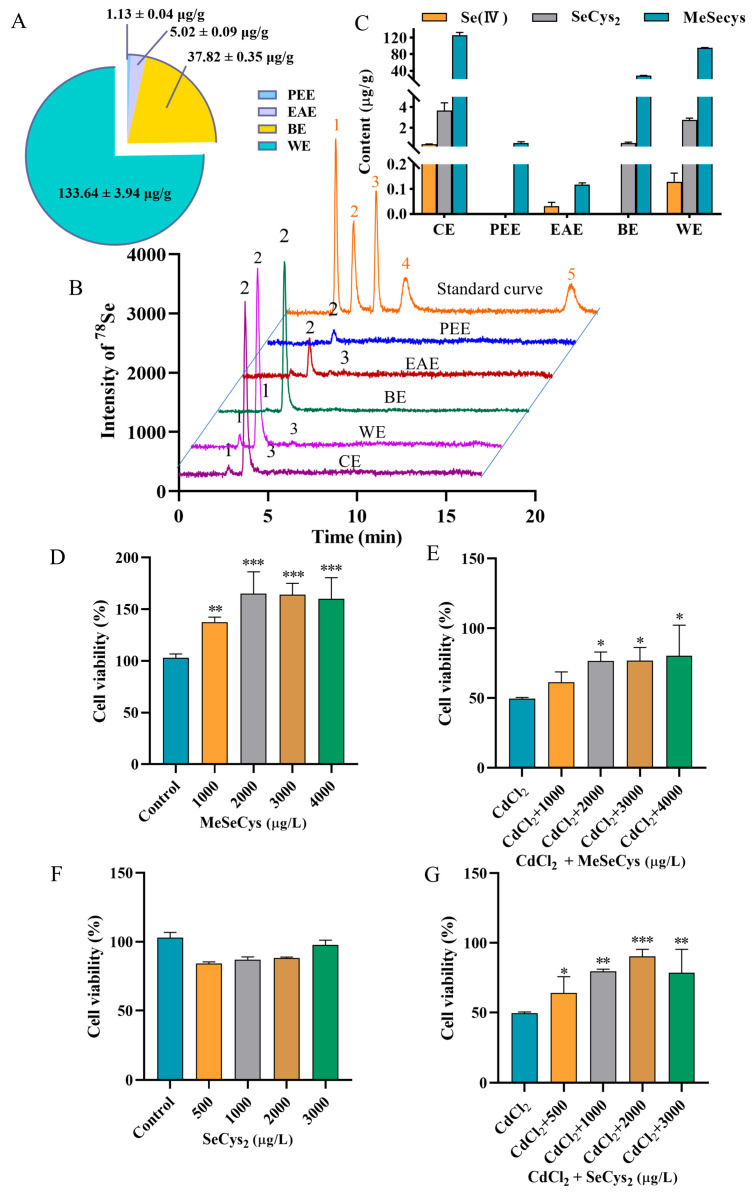
(**A**) Analytical results for total selenium content in PEE, EAE, BE, and WE. (**B**) HPLC chromatograms of 5 selenium species in standard solution and WE. Peaks of 1–5 represent SeCys_2_, MeSeCys, Se (IV), SeMet, and Se (VI)). (**C**) HPLC-ICP-MS results for selenium speciation in WE. The viability of cells treated with MeSeCys alone (**D**) and co-treated with MeSeCys and CdCl_2_ (**E**). The viability of cells treated with SeCys_2_ alone (**F**) and co-treated with SeCys_2_ and CdCl_2_ (**G**). For (**D**), ** *p* < 0.01, and *** *p* < 0.001 vs. control group. For (**E**,**G**), * *p* < 0.05, ** *p* < 0.01, and *** *p* < 0.001 vs. CdCl_2_ group.

Recent studies have demonstrated that rapeseed has a unique ability to enrich selenium and can quickly absorb inorganic selenium and convert it into organic selenium [20]. To investigate the influence of extraction processes on the total selenium content in different fractions, the four fraction samples were introduced into the ICP-MS system after acid digestion. As shown in Figure 3A, the total selenium contents in PEE, EAE, BE, and WE were found to be 1.13 ± 0.04, 5.02 ± 0.09, 37.82 ± 0.35, and 133.64 ± 3.94 µg/g, respectively. To further analyze the selenium species in different fractions, the five selenium species (Se (IV), Se (VI), SeCys_2_, MeSeCys, and SeMet) were separated and quantificationally detected using HPLC-ICP-MS. The chromatograms and speciation analysis results are shown in Figure 3B,C, respectively. Neither SeMet nor Se(VI) was detected in any fraction. MeSeCys was identified as the dominant species, with trace amounts of SeCys_2_ and Se(IV) detected in CE. PEE contained only MeSeCys (0.56 ± 0.08 µg/g), and EAE exhibited low levels of MeSeCys (0.12 ± 0.006 µg/g) and Se(IV) (0.03 ± 0.001 µg/g). Additionally, BE contained MeSeCys (28.21 ± 0.68 µg/g) with minor SeCys_2_ (0.54 ± 0.07 µg/g). WE contained MeSeCys (95.59 ± 0.90 µg/g), Se(IV) (0.13 ± 0.03 µg/g), and SeCys_2_ (2.75 ± 0.20 µg/g). The results revealed that the organic selenium species were mainly retained in the WE fraction, which was also found to significantly protect the cells against CdCl_2_-induced toxicity. Based on the results of both the cell viability assessment and the selenium speciation analysis, the WE fraction was chosen for subsequent experiments.

Subsequently, the positive effects of MeSeCys and SeCys_2_ against Cd-induced damage in TM4 cells were evaluated (Figure 3D–G). Treatment with MeSeCys alone significantly enhanced TM4 cell viability, with cell viability rates of 137.50%, 165.00%, 164.00%, and 160.00%, respectively. In contrast, SeCys_2_ did not exhibit a significant effect on TM4 cell viability. Compared to the CdCl_2_ group, co-exposure with 2000–4000 µg/L MeSeCys markedly mitigated Cd-induced damage, with cell viability of 76.67–80.33%. Furthermore, co-exposure with 500–3000 µg/L SeCys_2_ also attenuated Cd toxicity, resulting in cell viability of 64.00–90.33%. In summary, both MeSeCys and SeCys_2_ present in WE significantly alleviated Cd-induced cytotoxicity in TM4 cells.

### 3.4. RNA-Seq Analysis of the Alleviation by WE of CdCl_2_-Induced Toxicity in TM4 Cells

**Figure 4:**
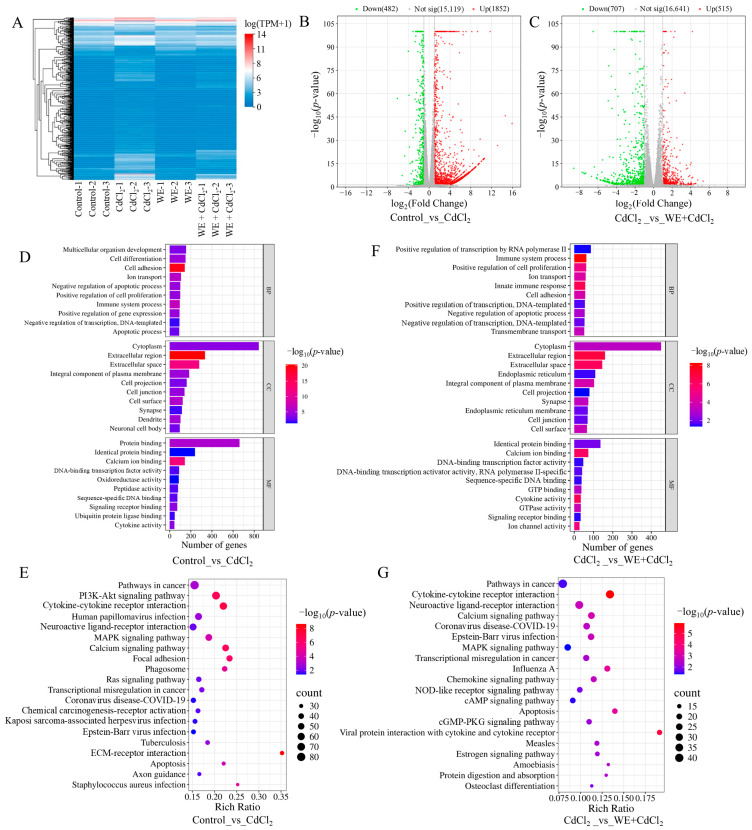
RNA sequencing analysis. (**A**) Sample clustering analysis. (**B**) Comparison of volcano plot of DEGs in control vs. CdCl_2_ group. (**C**) Comparison of volcano plot of DEGs in CdCl_2_ group vs. WE + CdCl_2_ group. (**D**) GO enrichment analysis of DEGs in control vs. CdCl_2_ group. (**E**) KEGG pathway enrichment analysis of DEGs in control vs. CdCl_2_ group. (**F**) GO enrichment analysis of DEGs in CdCl_2_ group vs. WE + CdCl_2_ group. (**G**) KEGG pathway enrichment analysis of DEGs in CdCl_2_ group vs. WE + CdCl_2_ group.

To further investigate the mechanisms behind the alleviation by WE of the cell injury caused by CdCl_2_, RNA-seq analysis was conducted. As shown in Figure 4A, sample clustering analysis showed that the RNA-seq results were of high accuracy and reproducibility. Compared to the control group, 2334 differentially expressed genes (DEGs) were obtained in the CdCl_2_ group (Figure 4B). Subsequently, gene ontology (GO) analysis was conducted on those DEGs. As shown in Figure 4D, biological processes were primarily related to cell differentiation, ion transport, and apoptotic process; cellular components were mainly associated with the cytoplasm, extracellular space, and plasma membrane; and molecular functions were predominantly associated with protein binding, oxidoreductase activity, and cytokine activity. Furthermore, the Kyoto encyclopedia of genes and genomes (KEGG) analysis results indicated that the signaling pathways included the PI3K-Akt pathway, MAPK pathway, and apoptosis (Figure 4E). Previous research demonstrated that Cd exposure could cause testicular cell apoptosis by activating the MAPK pathway [27].

Additionally, 1222 DEGs were identified in the WE + CdCl_2_ group compared to the CdCl_2_ control, consisting of 515 upregulated and 707 downregulated genes (Figure 4C). Subsequently, GO and KEGG analysis were conducted on these 1222 DEGs (Figure 4F,G). As shown in Figure 4F, the biological processes were mainly related to cell proliferation and apoptotic processes; cellular components were associated with the cytoplasm, extracellular space, and plasma membrane; and molecular functions were predominantly linked to protein binding, calcium ion binding, and cytokine activity. Furthermore, the top 20 signaling pathways obtained via KEGG analysis are presented in Figure 4G, including cytokine–cytokine receptor interaction, the MAPK pathway, and apoptosis. It is worth noting that the MAPK pathway was notably enriched. As a result, we aimed to further experimentally validate the MAPK pathway and apoptosis.

### 3.5. Effects of WE on CdCl_2_-Induced Oxidative Stress in TM4 Cells

**Figure 5:**
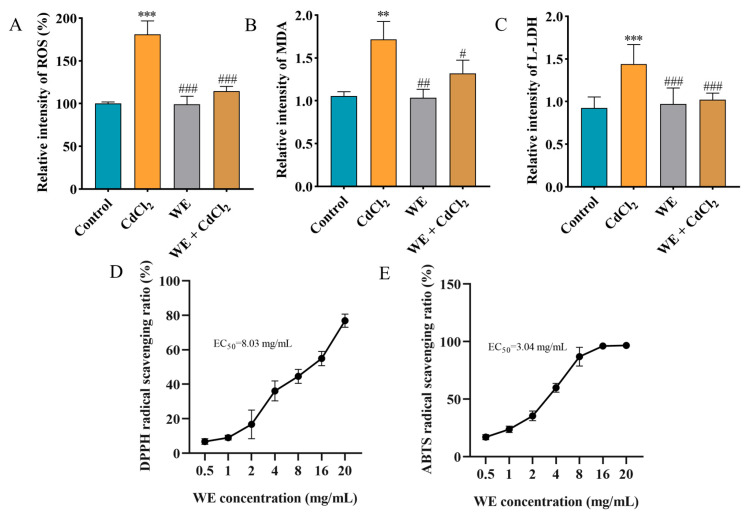
The antioxidant capacity of WE. (**A**) ROS level. (**B**) MDA level. (**C**) L-LDH level. (**D**,**E**) Determination of DPPH and ABTS free radical scavenging activity of WE. ** *p* < 0.01, and *** *p* < 0.001 vs. control group. ^#^ *p* < 0.05, ^##^ *p* < 0.01, and ^###^ *p* < 0.001 vs. CdCl_2_ group.

Studies have reported that the MAPK pathway could be activated by ROS, and Cd exposure may cause oxidative stress, subsequently leading to cell apoptosis [28]. Consequently, we determined the oxidative stress levels in TM4 cells exposed to CdCl_2_ and assessed whether WE intervention could mitigate CdCl_2_-induced cell damage by modulating oxidative stress. Firstly, the intracellular antioxidant activity of WE against CdCl_2_-induced ROS generation in TM4 cells was examined (Figure 5A). The ROS level dramatically increased to 181% in the cells exposed to CdCl_2_ compared to the control group. Conversely, the intracellular ROS level was notably reduced in the WE + CdCl_2_ group compared to the CdCl_2_ group, suggesting that WE could observably reduce ROS formation caused by CdCl_2_. Furthermore, we also determined the levels of the oxidative stress-related indicator MDA [29]. As shown in Figure 5B, the MDA level in cells exposed to CdCl_2_ was 1.72 times higher than that in the control group, while WE intervention significantly decreased the MDA level. Additionally, the LDH level, commonly regarded as a biomarker for cell damage [30], was increased in the CdCl_2_ group compared to the control group, and WE intervention notably reduced the L-LDH level (Figure 5C). Taken together, these results suggest that CdCl_2_ treatment significantly causes oxidative stress in TM4 cells, whereas WE exhibits a significant alleviating effect on oxidative damage caused by CdCl_2_. Moreover, we also evaluated the extracellular antioxidant activity of WE, and the results indicate that the EC50 for DPPH and ABTS radicals was 8.03 and 3.04 mg/mL, respectively, demonstrating the good free radical scavenging capabilities of WE (Figure 5D,E).

### 3.6. Effects of WE on Cell Cycle, Inflammation, and Apoptosis of TM4 Cells Under CdCl_2_ Exposure

**Figure 6:**
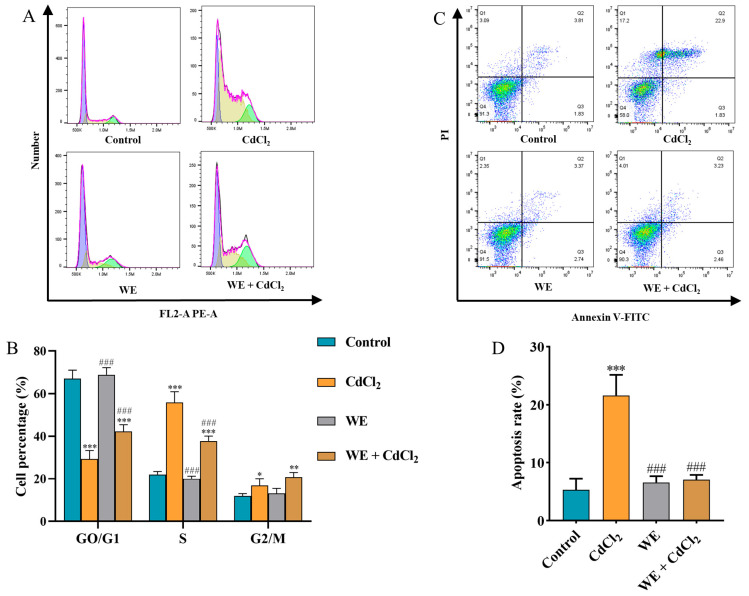
Flow cytometry analysis. (**A**,**B**) The cell cycle distribution of TM4 cells treated with CdCl_2_, WE, and WE + CdCl_2_. (**C**,**D**) The cell apoptosis of TM4 cells treated with CdCl_2_, WE, and WE + CdCl_2_. * *p* < 0.05, ** *p* < 0.01, and *** *p* < 0.001 vs. control group. ^###^ *p* < 0.001 vs. CdCl_2_ group.

Evidence indicates that ROS accumulation can lead to cell cycle arrest, inflammation, and apoptosis [31]. Therefore, we explored whether the mechanism behind WE alleviating cell damage caused by CdCl_2_ was associated with cell cycle progression. Compared to the control group, the cell number in the GO/G1 phase notably reduced, whereas the cell number in the S and G2/M phase significantly increased in the CdCl_2_ group, suggesting that CdCl_2_ exposure triggered cell cycle arrest in TM4 cells. In contrast, WE intervention increased the percentage of GO/G1-phase cells and lowered the percentage of S-phase cells, suggesting that WE intervention alleviated cell cycle arrest caused by CdCl_2_ (Figure 6A,B). Meanwhile, the cell apoptosis rate was significantly increased in the CdCl_2_ group compared to the control group. After WE intervention, the apoptosis rate of TM4 cells was significantly reduced (Figure 6C,D).

**Figure 7:**
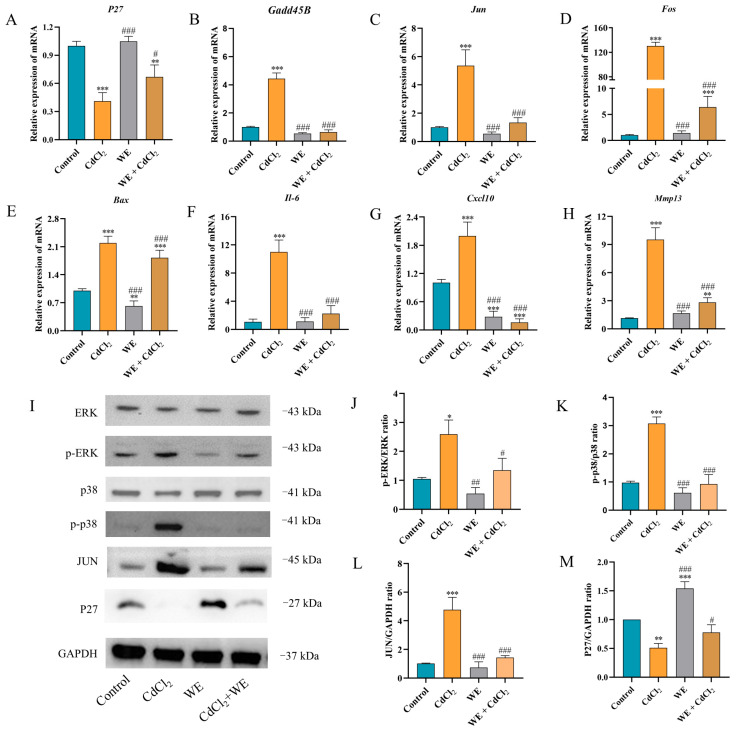
RT-qPCR and Western blot analysis. (**A**–**H**) The mRNA expression levels of *p27*, *Gadd45B*, *Jun*, *Fos*, *Bax*, *Il-6*, *Cxcl10*, and *Mmp13* in TM4 cells. (**I**–**M**) The expression levels of ERK, p38, JUN, and P27 proteins in TM4 cells. * *p* < 0.05, ** *p* < 0.01, and *** *p* < 0.001 vs. control group. ^#^ *p* < 0.05, ^##^ *p* < 0.01, and ^###^ *p* < 0.001 vs. CdCl_2_ group.

To further explore the mechanisms behind the effects of WE against CdCl_2_-induced cell damage, the expression of several key genes (*P27*, *Gadd45B*, *Cxcl10*, *Mmp13*, *Il-6*, *Bax*, *Fos*, and *Jun*) related to cell cycle regulation, inflammation, and apoptosis was assessed via qRT-PCR according to RNA-seq data. Compared to the control group, CdCl_2_ treatment resulted in the downregulation of *P27* gene expression, whereas WE intervention led to the upregulation of *P27* expression (Figure 7A). Furthermore, CdCl_2_ exposure significantly elevated the mRNA expression level of *Gadd45B*, a gene associated with cell cycle arrest and apoptosis, while WE intervention diminished *Gadd45B* expression (Figure 7B). These results suggest that WE may alleviate the cell cycle arrest induced by CdCl_2_ exposure. According to Figure 7C–H, CdCl_2_ exposure notably upregulated the expression levels of inflammation-related genes (*Cxcl10*, *Mmp13*, and *Il-6*) and apoptotic-related genes (*Bax*, *Fos*, and *Jun*), while WE intervention significantly lowered the mRNA expressions of these genes. Collectively, our findings suggest that WE may mitigate CdCl_2_-induced cell damage by alleviating cell cycle arrest and inhibiting both inflammation and apoptosis.

### 3.7. Effects of WE on MAPK Signaling Pathway After CdCl_2_ Exposure

The phosphorylation of MAPK plays crucial roles in modulating the inflammatory response triggered by Cd [32]. As shown in Figure 7I–M, compared to the control group, CdCl_2_ treatment markedly increased the phosphorylation levels of ERK and p38 protein in TM4 cells, as well as the protein expression of JUN. Conversely, the level of P27 protein was markedly reduced following CdCl_2_ exposure. WE intervention notably reduced the JUN protein level and the phosphorylation level of ERK and p38 induced by CdCl_2_ exposure and increased P27 protein expression. The results suggest that WE intervention could protect TM4 cells from CdCl_2_-induced damage by inhibiting the activation of the ERK/p38 MAPK pathway.

### 3.8. RNA-Seq Analysis of MeSeCys and SeCys_2_ to Determine Mechanisms Alleviating CdCl_2_-Induced Toxicity in TM4 Cells

**Figure 8:**
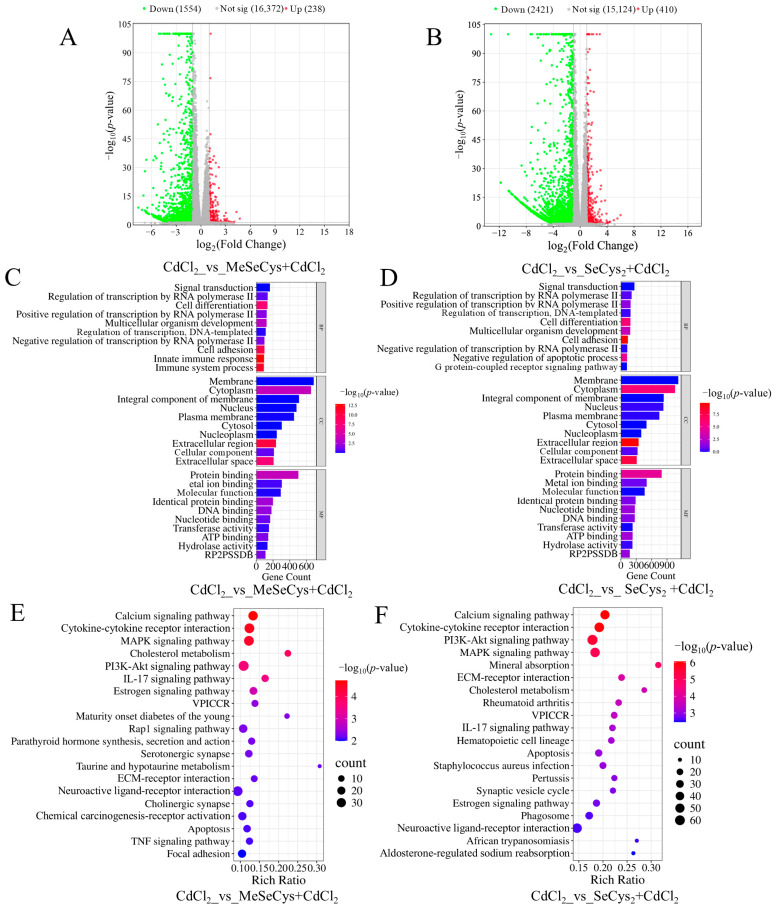
(**A**) Comparison of volcano plot of DEGs in CdCl_2_ group vs. MeSeCys + CdCl_2_ group. (**B**) Comparison of volcano plot of DEGs in CdCl_2_ group vs. SeCys_2_ + CdCl_2_ group. (**C**) GO enrichment analysis of the DEGs in CdCl_2_ group vs. MeSeCys + CdCl_2_ group. (**D**) GO enrichment analysis of DEGs in CdCl_2_ group vs. SeCys_2_ + CdCl_2_ group. (**E**) KEGG pathway enrichment analysis of DEGs in CdCl_2_ group vs. MeSeCys + CdCl_2_ group. (**F**) KEGG pathway enrichment analysis of DEGs in CdCl_2_ group vs. SeCys_2_ + CdCl_2_ group.

To determine the mechanisms by which the two organic selenium species (MeSeCys and SeCys_2_) alleviated CdCl_2_-induced damage in TM4 cells, RNA-seq analysis was also conducted. As shown in Figure 8A,B, compared with the CdCl_2_ group, 1792 DEGs were identified in the MeSeCys + CdCl_2_ group, whereas 2831 DEGs were identified in the SeCys_2_ + CdCl_2_ group. Subsequently, GO and KEGG analysis were conducted (Figure 8C–F). The top 20 enriched signaling pathways for the MeSeCys + CdCl_2_ group and SeCys_2_ + CdCl_2_ group are shown in Figure 8E,F, respectively. The results showed that the signaling pathways modulated by both treatments largely overlapped and involved the MAPK signaling pathway, the P13K-Akt signaling pathway, cytokine–cytokine receptor interaction, the calcium signaling pathway, the IL-17 signaling pathway, and apoptosis. Notably, the MAPK signaling pathway exhibited significant enrichment in both groups, consistent with observations in the WE + CdCl_2_ group.

### 3.9. Effects of MeSeCys and SeCys_2_ on Oxidative Stress, Cell Cycle, Inflammation, and Apoptosis in TM4 Cells Under CdCl_2_ Exposure

**Figure 9:**
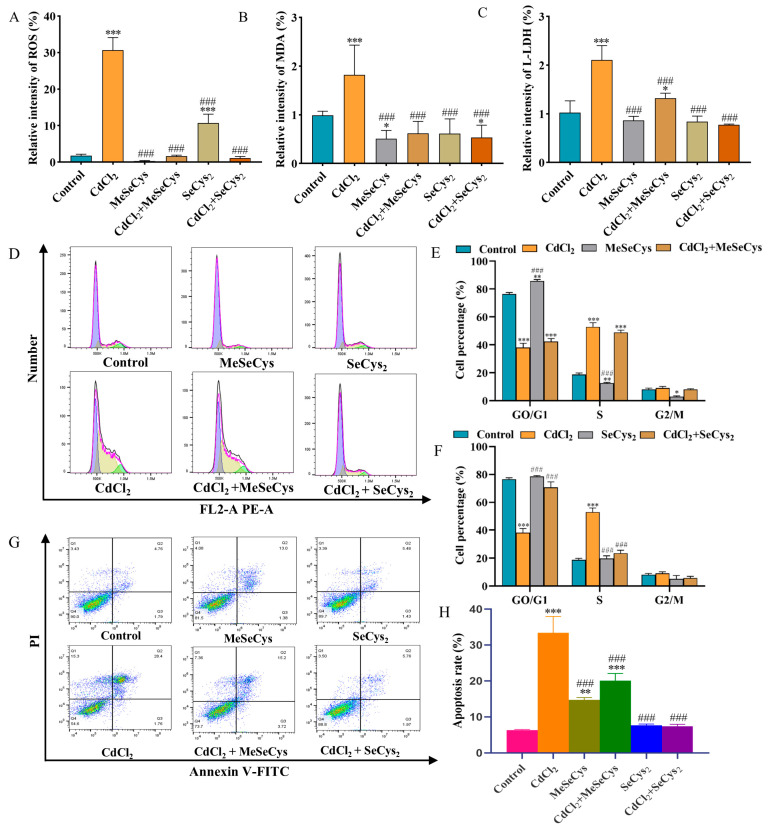
(**A**) ROS level. (**B**) MDA level. (**C**) L-LDH level. (**D**–**F**) Cell cycle. (**G**,**H**) Cell apoptosis. * *p* < 0.05, ** *p* < 0.01, and *** *p* < 0.001 vs. control group. ^###^ *p* < 0.001 vs. CdCl_2_ group.

As illustrated in Figure 9A–C, compared with the CdCl_2_ group, the levels of ROS, MDA, and L-LDH were significantly reduced in both the MeSeCys + CdCl_2_ and SeCys_2_ + CdCl_2_ group, demonstrating that MeSeCys and SeCys_2_ could mitigate CdCl_2_-induced oxidative stress damage in TM4 cells. Figure 9D–F reveal that SeCys_2_ intervention increased the percentage of G0/G1-phase cells in TM4 cells by 32.53% while reducing the percentage of S-phase cells by 29.43%. Conversely, MeSeCys intervention did not significantly alter the distribution of cells in these phases. The apoptosis rate of TM4 cells was reduced by 13.36% and 26.01% in the MeSeCys + CdCl_2_ group and SeCys_2_ + CdCl_2_ group, respectively, indicating that MeSeCys and SeCys_2_ could inhibit CdCl_2_-induced apoptosis (Figure 9G,H).

**Figure 10:**
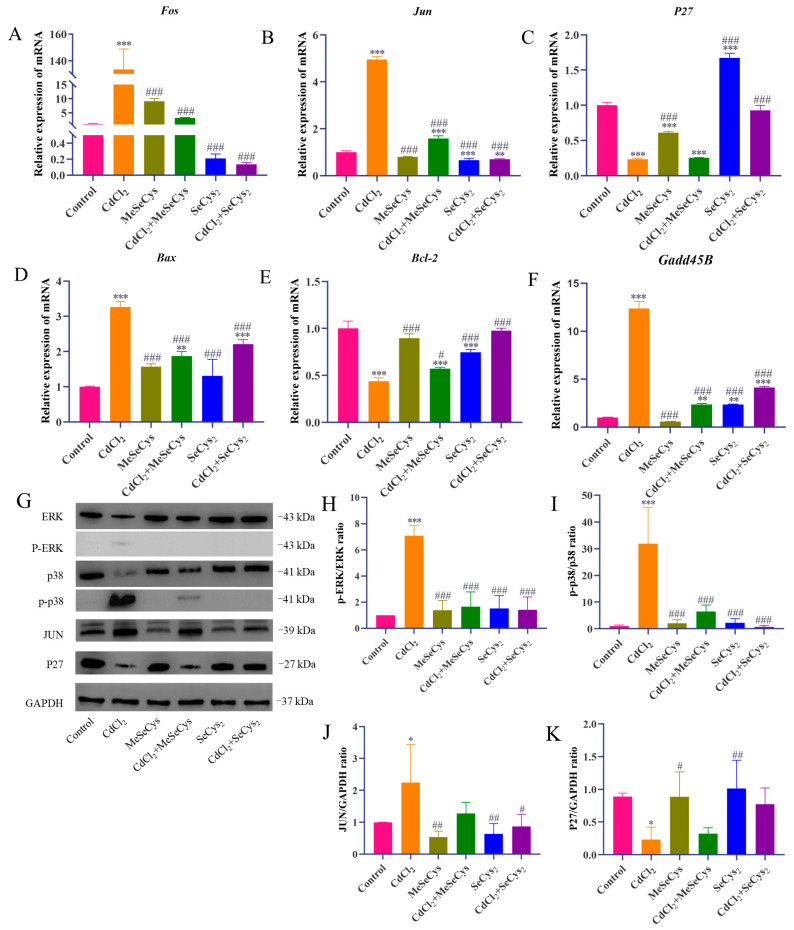
RT-qPCR and Western blot analysis. (**A**–**F**) The mRNA expression levels of *Fos*, *Jun*, *p27*, *Bax*, *Bcl-2*, and *Gadd45B* in TM4 cells. (**G**–**K**) The expression levels of ERK, p38, JUN, and P27 proteins in TM4 cells. * *p* < 0.05, ** *p* < 0.01, and *** *p* < 0.001 vs. control group. ^#^ *p* < 0.05, ^##^ *p* < 0.01, and ^###^ *p* < 0.001 vs. CdCl_2_ group.

The mRNA expression levels of key genes (*Fos*, *Jun*, *Bax*, *Bcl-2*, *P27*, and *Gadd45B*) were assessed (Figure 10A–F). Compared with the CdCl_2_ group, the mRNA expression of *Jun*, *Fos*, *Bax*, and *Gadd45B* was significantly downregulated, whereas the mRNA expression of *P27* and *Bcl-2* was upregulated in the MeSeCys + CdCl_2_ group and SeCys_2_ + CdCl_2_ group. To further elucidate whether MeSeCys and SeCys_2_ could reduce CdCl_2_-induced TM4 cell damage via the MAPK pathway, Western blotting was performed. Compared with the CdCl_2_ group, the protein level of JUN and the phosphorylation levels of ERK and p38 in the SeCys_2_ + CdCl_2_ group were significantly decreased, as were the phosphorylation levels of ERK and p38 in the MeSeCys + CdCl_2_ group (Figure 10G–K).

## 4. Discussion

Since the 1950s, the global prevalence of infertility has been steadily rising, and male factors contribute to approximately 40–50% of infertility cases [33]. Cd, as one of the main environmental pollutants, poses a substantial threat to the male reproductive system, for example, by causing testicular damage, bleeding, edema, atrophy, necrosis, and a decrease in sperm quality [34]. Within various experimental models, TM4 cells represent a critical cellular component of the spermatogenic epithelium, playing an essential role in spermatogenesis [22]. Notably, different selenium species have been demonstrated to significantly mitigate Cd-induced reproductive toxicity [8].

Research has indicated that rapeseed is abundant in a diverse range of nutrients that are beneficial to human health [16]. In recent years, it has been discovered that edible rapeseed sprouts not only contain vitamin C, minerals, and dietary fiber but also exhibit a pronounced capacity for selenium enrichment [20]. This study is the first to discover that selenium-enriched rapeseed flowering stalks significantly attenuate Cd-induced cytotoxicity in TM4 cells. The extraction efficacy of bioactive components is closely influenced by parameters such as the solvent type, solvent concentration, solid–liquid ratio (*v*/*w*), and extraction time [35]. Through single-factor optimization experiments, the CE of selenium-enriched rapeseed flowering stalks obtained under the conditions of 50% methanol concentration, a solid–liquid ratio of 1:15, and an extraction time of 9 min proved to be significantly more effective in alleviating Cd-induced cytotoxicity.

Bioassay-guided fractionation techniques have been widely utilized to identify the most bioactive fraction in various foods based on specific biochemical assays [36]. For instance, Daud et al. identified the most bioactive fraction in *Artocarpus heterophyllus* L. J33 variety fruit waste via a bioassay-guided fractionation approach based on a DPPH assay [37]. To further identify the effective bioactive components in selenium-enriched flowering stalks, in this study, we obtained PEE, EAE, BE, and WE from CE. The extraction yield of WE was the highest (76.65 ± 2.56%), while the extraction yields of PEE, EAE, and BE were only 0.83 ± 0.11%, 1.68 ± 0.51%, and 12.96 ± 1.56%, respectively. Importantly, compared with PEE, EAE, and BE, WE significantly protected TM4 cells against Cd-induced cytotoxicity, achieving a cell viability rate of 127.99%. Subsequently, the contents of total selenium and different selenium species in the fractions (CE, PEE, EAE, BE, and WE) were determined via (HPLC-)ICP-MS. The results indicated that selenium was mainly concentrated in WE, and the main selenium species in WE was MeSeCys (95.59 ± 0.90 µg/g). In addition, small amounts of SeCys_2_ (2.75 ± 0.20 µg/g) and Se(IV) (0.13 ± 0.03 µg/g) were also detected in WE. Cellular assays demonstrated that both MeSeCys and SeCys_2_ significantly mitigated Cd-induced cytotoxicity in TM4 cells. Previous studies have shown that MeSeCys can ameliorate di-(2-ethylhexyl)phthalate-induced ferroptosis in testicular Sertoli cells via the activation of the Nrf2/GPX4 signaling pathway [38], while SeCys_2_ can significantly reduce the toxicity of cadmium and mercury and improve sperm motility and oxygen consumption [39].

Transcriptomics has emerged as a pivotal approach to exploring the mechanisms underlying the effects of functional foods [40]. In this study, WE, MeSeCys, and SeCys_2_ intervention modulated apoptotic signaling pathways, MAPK signaling pathways, and cytokine–cytokine receptor interactions based on transcriptomic techniques. Research has indicated that Cd exposure can cause testicular cell damage through the MAPK signaling pathway [27], and the activation of this pathway by ROS plays a crucial role in the initiation of cell differentiation, cell cycle, and apoptosis [41]. Moreover, Cd exposure has been demonstrated to induce cell death through mechanisms involving oxidative damage, cell cycle arrest, inflammation, and apoptosis [42]. MDA serves as a principal biomarker of oxidative cellular injury [25], and L-LDH is a constitutive enzyme released upon cellular damage [43]. Studies have shown that Cd exposure increases the contents of MDA and L-LDH in cells [6]. Our findings demonstrate that WE, MeSeCys, and SeCys_2_ intervention significantly attenuates ROS levels and inhibits the release of MDA and L-LDH in TM4 cells, thereby mitigating Cd-induced oxidative stress damage. DPPH and ABTS assays, widely recognized for their usefulness in evaluating antioxidant capacity, were employed to assess free radical scavenging activity [44]. This study revealed that WE exhibits substantial scavenging activity against DPPH and L-LDH radicals. The accumulation of ROS is known to precipitate cell cycle arrest, inflammation, and apoptosis [31]. Flow cytometry analyses indicated that Cd exposure induced cell cycle arrest and apoptosis in TM4 cells, whereas co-treatment with WE, MeSeCys, and SeCys_2_ alleviated these Cd-induced effects.

Previous studies have reported that Cd exposure alters the expression of genes such as *Cxcl10*, *Mmp13*, *Il-6*, *Bcl-2*, *Bax*, *Fos*, and *Jun*, thereby promoting inflammation and apoptosis [45,46]. Additionally, *P27* and *Gadd45B* have been identified as key regulators of cell cycle progression [47,48]. In this study, it was demonstrated that WE, MeSeCys, and SeCys_2_ inhibited the phosphorylation of ERK and p38 and modulated the expression of Cd-induced genes including *Jun*, *Fos*, *Bax*, *Bcl-2*, *P27*, and *Gadd45B*, as well as the proteins JUN and P27, ultimately suppressing Cd-induced apoptosis in TM4 cells.

## 5. Conclusions

In this study, for the first time, a bioactivity-guided approach was used to confirm that the potent cytoprotective effect of selenium-enriched rapeseed flowering stalks against CdCl_2_-induced cytotoxicity is primarily associated with its WE fraction, which was found to be enriched with the bioactive selenium compound (MeSeCys and SeCys_2_). Moreover, we elucidated the mechanisms through which WE, MeSeCys, and SeCys_2_ alleviate CdCl_2_-induced cell damage via RNA-seq, qPCR, and Western blot methods. Our results indicate that their mechanisms are potentially associated with a reduction in oxidative damage, the inhibition of the ERK/p38 MAPK signaling pathway, and the suppression of cell cycle arrest, inflammation, and apoptosis in TM4 cells caused by CdCl_2_ exposure. However, we acknowledge certain limitations, particularly the need for further elucidation of the precise active compounds in WE that reduced CdCl_2_-induced cytotoxicity. Despite this limitation, our results provide strong experimental evidence and a theoretical foundation for the development of selenium-enriched rapeseed in functional foods.

## Data Availability

The original contributions presented in this study are included in the article/Supplementary Materials. Further inquiries can be directed to the corresponding authors.

## Supplementary Materials

The following supporting information can be downloaded at https://www.mdpi.com/article/10.3390/antiox14111297/s1, Figure S1: Cell viability of TM4 cells exposed to CdCl_2_ with different incubation concentrations for 24 h; Figure S2: Effects of different extraction conditions on the viability of TM4 cells based on DNA assay. The effects of methanol concentration on the cell viability treated with CE alone (A) and co-treated with CE and CdCl_2_ (B). The effects of solid-liquid ratio on the cell viability treated with CE alone (C) and co-treated with CE and CdCl_2_ (D). The effects of extraction time on the cell viability treated with CE alone (E) and co-treated with CE and CdCl_2_ (F); Figure S3: (A) The effects of PEE, EAE, BE and WE on the cell viability based on DNA assay. (B) The effects of PEE, EAE, BE and WE on Cd-induced cytotoxicity in TM4 cells based on DNA assay; Table S1: Parameters and their levels in the single-factor experiments; Table S2: Primer sequences for qRT-PCR.

## Abbreviations

The following abbreviations are used in this manuscript:

Cd	Cadmium
MeSeCys	Methylselenocysteine
SeCys_2_	Selenocystine
SeMet	Selenomethionine
TM4 cells	Mouse Sertoli cells
CdCl_2_	Cadmium chloride
CE	Crude extract
PEE	Petroleum ether extract
EAE	Ethyl acetate extract
BE	n-butanol extract
WE	Water extract
DPPH	2,2-diphenyl-1-picrylhydrazyl
ABTS	2,2′-azino-bis-3-ethylbenzthiazoline-6-sulphonate
ROS	Reactive oxygen species
MAD	Malonic dialdehyde
L-LDH	L-lactate dehydrogenase
CCK-8	Cell counting kit-8
MAPK	Mitogen-activated protein kinase
GAPDH	Glyceraldehyde-3-phosphate dehydrogenase
GO	Gene ontology
KEGG	Kyoto encyclopedia of genes and genomes
HPLC-ICP-MS	High-performance liquid chromatography–inductively coupled plasma mass spectrometry
DEGs	Differentially expressed genes
