# Supplementary material for: Investigation into Bioactive Selenium Species and the Mechanism of Action Behind Selenium-Enriched Rapeseed Flowering Stalks Alleviating Cadmium-Induced Toxicity in Mouse Sertoli Cells
Source: Antioxidants (Basel). 2025 Oct 28;14(11):1297. doi: 10.3390/antiox14111297 (PMC12649212; doi:10.3390/antiox14111297)
Supplement: Supplementary file 1 [file antioxidants-14-01297-s001.zip › antioxidants-3872663-supplementary.pdf]

Investigation of the Bioactive Selenium Species and Mechanism of Selenium-Enriched Rapeseed Flowering Stalks in Alleviating Cadmium-Induced Toxicity in Mouse Sertoli Cells

Huatao Che [1], Yiqing Lu [1,2], Tong Li [1], Xiaoli Fang [1], Xinfa Wang [1,3], Hanzhong Wang [1,3], Xiaoling Dun [1,*] and Zhenna Chen [1,*]

[1] Key Laboratory of Biology and Genetic Improvement of Oil Crops, Oil Crops Research Institute of the Chinese Academy of Agricultural Sciences, Ministry of Agriculture and Rural Affairs, Wuhan 430062, China
[2] Institute of Crop Science, Zhejiang University, Hangzhou 310058, China
[3] Hubei Hongshan Laboratory, Wuhan 430070, China
* Correspondence: dunxiaoling@caas.cn (X.D.); chenzhenna@caas.cn (Z.C.)

Contents:

Figure S1. Cell viability of TM4 cells exposed to $CdCl_2$ with different incubation concentrations for 24 h.

Figure S2. Effects of different extraction conditions on the viability of TM4 cells based on DNA assay. The effects of methanol concentration on the cell viability treated with CE alone (**A**) and co-treated with CE and $CdCl_2$ (**B**). The effects of solid-liquid ratio on the cell viability treated with CE alone (**C**) and co-treated with CE and $CdCl_2$ (**D**). The effects of extraction time on the cell viability treated with CE alone (**E**) and co-treated with CE and $CdCl_2$ (**F**). The results were expressed as mean ± SD (n = 3). For (**A**,**C**,**E**) * $p < 0.05$, ** $p < 0.01$, *** $p < 0.001$ vs. control group. For (**B**,**D**,**F**) * $p < 0.05$, ** $p < 0.01$, *** $p < 0.001$ vs. $CdCl_2$ group.

Figure S3. (**A**) The effects of PEE, EAE, BE and WE on the cell viability based on DNA assay. (**B**) The effects of PEE, EAE, BE and WE on Cd-induced cytotoxicity in TM4 cells based on DNA assay. The results were expressed as mean ± SD (n = 3). For (**A**) * $p < 0.05$, ** $p < 0.01$, *** $p < 0.001$ vs. control group. For (**B**) * $p < 0.05$, ** $p < 0.01$, *** $p < 0.001$ vs. $CdCl_2$ group.

Table S1. Parameters and their levels in the single-factor experiments.

Table S2. Primer sequences for qRT-PCR.

Optimization of extraction conditions

In this work, single-factor experiments were conducted to study the effect of methanol concentration (30%-90%), solid-liquid ratio (1:10-1:25 (w/v) (g/mL)) and extraction time (6-18 min) on the efficacy of the obtained crude extract (CE) in mitigating CdCl2-induced cytotoxicity. All extractions were performed using an ultrasonic cleaner operating at a power of 300 W and a frequency of 40 kHz, maintaining a constant temperature of 30°C. Firstly, the influence of methanol concentration ranging from 30% to 90% was investigated using a fixed solid-to-liquid ratio of 1:15 and an extraction time of 12 min. As can be seen in Figure 1A,B, a methanol concentration of 50% was used in subsequent experiments. Subsequently, the effect of solid-liquid ratio in the range of 1:10-1:25 (g/mL) on the cell viability was examined while maintaining a 50% methanol concentration and a 12 min extraction time. As illustrated in Figure 1C,D, the optimal solid-liquid ratio was selected as 1:15 in subsequent experiments. Finally, the effect of extraction time on the cell viability was investigated in the range of 6-18 min, with methanol concentration and solid-liquid ratio fixed at 50% and 1:15. As can be seen from Figure 1E,F, the optimal extraction time was chosen as 9 min. In short, the following experimental conditions were used: (a) methanol concentration: 50%; (b) solid-liquid ratio: 1:15; (c) extraction time: 9 min.

(HPLC-)ICP-MS analysis

For the detection of total selenium content, 20 mg of freeze-dried extracts and 2 mL of nitric acid were added into DigiBlock (LabTech, Beijing, China). The solution was digested at 85°C for 3 h, and then evaporated at 120°C to nearly dryness. The obtained residue was dissolved in 3 mL deionized water and introduced into ICP-MS detection. For selenium species analysis, 20 mg extracts, 2 mL protease XIV (10 mg/mL) and 18 mL ultrapure water were added into a plastic centrifuge tube. Following a 40-min ultrasound treatment, the supernatant was obtained through centrifugation (12000 rpm×15 min) and introduced into HPLC-ICP-MS for the seperation and detection of different selenium species.

Cell culture

TM4 cells were obtained from Procell Life Science & Technology Co., Ltd (Wuhan, China). TM4 cells were cultured in TM4 cell specific culture medium containing DMEM/F12 with 5% (v/v) HS, 2.5% (v/v) FBS and 1% P/S (v/v) at 37℃ in a humidified incubator containing 5% CO_2.

CCK-8 assay

TM4 cells were firstly seeded at a density of 1×10^4 cells per well in a 96-well plate for 24 h. Then, the medium was removed, and cells were treated with $CdCl_2$, CE, WE, PEE, EAE or BE for 24 h. Next, the cells

were cleaned by PBS for 3 times and treated with 100 μL of CCK-8 reagent for 40 min at 37°C. Finally, cell viability was detected as the absorbance at 450 nm by a microplate reader (Thermo scientific, Waltham, USA).

DNA assay

The CyQUANT™ NF cell proliferation assay kit was used for DNA assay following the manufacturer's instruction. TM4 cells were firstly seeded at a density of 1×10^4 cells per well in a 96-well plate for 24 h. Then, the medium was removed, and cells were treated with $CdCl_2$, CE, WE, PEE, EAE or BE for 24 h. Next, the cells were cleaned by PBS and treated with CyQUANT™ NF reagent for 50 min at 37°C. Finally, the fluorescence intensity was measured on a microplate reader (Thermo scientific, Waltham, USA) with a wavelength of 480 nm for excitation and 520 nm for emission.

Flow cytometry analysis

TM4 cells were seeded at a density of 5×10^5 cells per well in a 6-well plate for 24 h. Then, the cells were exposed to various treatments for 24 h, and the cells were collected and washed by PBS for three times. After centrifugation (2990 rpm×5 min), the cells were treated according to the instructions of the cell apoptosis assay kit and cell cycle assay kit, respectively. Finally, the cell cycle variation and the level of intracellular apoptosis were detected by flow cytometry (Beckman, CA, USA), and the data was analyzed by FlowJo software.

Western blot analysis

TM4 cells were lysed using RIPA solution, and the total protein was determined utilizing the bicinchoninic acid protein assay kit (Shanghai, China). Subsequently, proteins were subjected to electrophoresis for separation. After that, proteins were transferred to a PVDF membrane, which was then blocked with 5% skim milk for 1.5 h. The membranes were incubated with primary antibodies overnight. Afterward, the membranes were treated with secondary antibodies and washed with TBST buffer for 3 times. Finally, the immunoreactive protein bands were visualized using the superfemto ECL chemiluminescence kit reagents (Nanjing, China) and imaged using a ChemiDoc™ XRS+ System (Bio-rad, MP, USA).

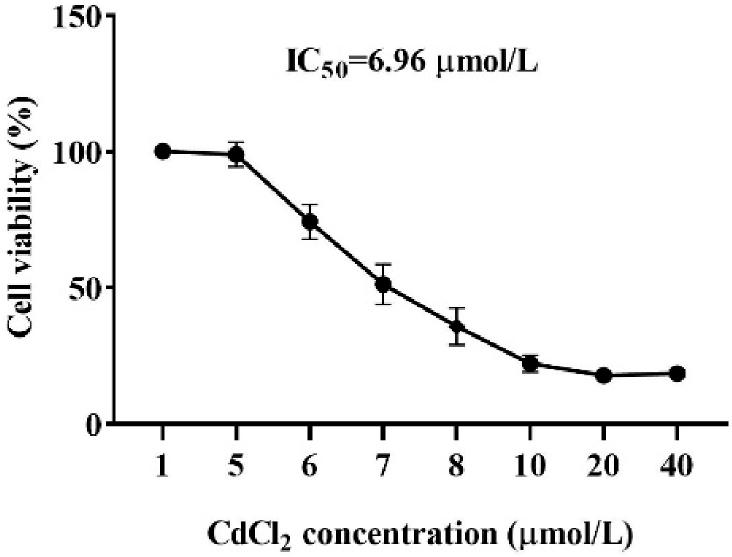

Figure S1. Cell viability of TM4 cells exposed to $CdCl_2$ with different incubation concentrations for 24 h.

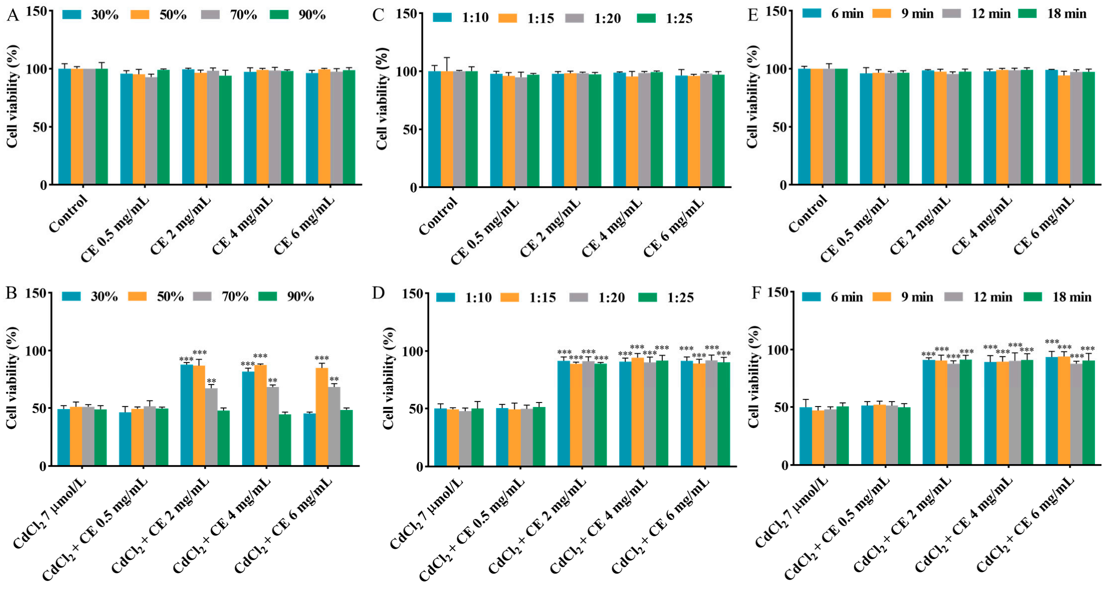

Figure S2. Effects of different extraction conditions on the viability of TM4 cells based on DNA assay. The effects of methanol concentration on the cell viability treated with CE alone (**A**) and co-treated with CE and $CdCl_2$ (**B**). The effects of solid-liquid ratio on the cell viability treated with CE alone (**C**) and co-treated with CE and $CdCl_2$ (**D**). The effects of extraction time on the cell viability treated with CE alone (**E**) and co-treated with CE and $CdCl_2$ (**F**). The results were expressed as mean ± SD (n = 3). For (**A**,**C**,**E**) * $p < 0.05$, ** $p < 0.01$, *** $p < 0.001$ vs. control group. For (**B**,**D**,**F**) * $p < 0.05$, ** $p < 0.01$, *** $p < 0.001$ vs. $CdCl_2$ group.

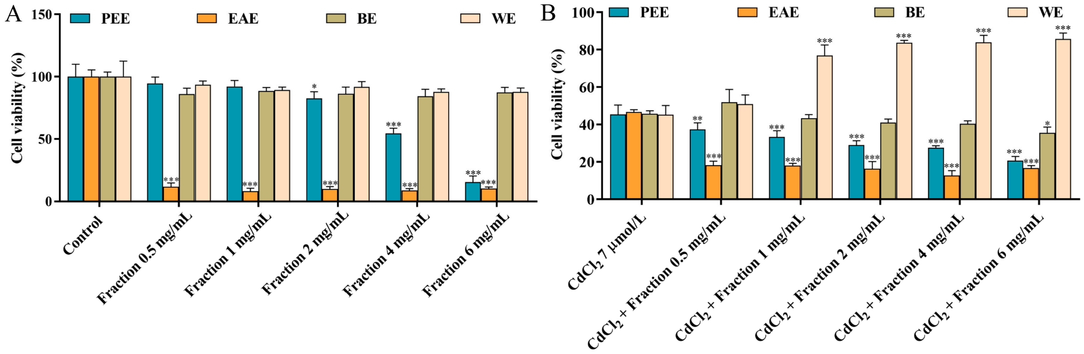

Figure S3. (**A**) The effects of PEE, EAE, BE and WE on the cell viability based on DNA assay. (**B**) The effects of PEE, EAE, BE and WE on Cd-induced cytotoxicity in TM4 cells based on DNA assay. The results were expressed as mean ± SD (n = 3). For (**A**) * $p < 0.05$, ** $p < 0.01$, *** $p < 0.001$ vs. control group. For (**B**) * $p < 0.05$, ** $p < 0.01$, *** $p < 0.001$ vs. $CdCl_2$ group.

Table S1. Parameters and their levels in the single-factor experiments.

Parameters	Methanol Concentration (%)	Solid-Liquid Ratio (g/mL)	Extraction Time (min)
Methanol concentration	30, 50, 70, 90	1:15	12
Solid-liquid ratio	50	1:10, 1:15, 1:20, 1:25	12
Extraction time	50	1:15	6, 9, 12, 18
Optimal extraction conditions	50	1:15	9

Table S2. Primer sequences for qRT-PCR.

Gene	Primer Sequence	Accession No.	Product Size (bp)
Gadd45B	F: CAATCTTCTTTTTACCCCTA<br>R: CAAAGTACAAGTCCAGATAC	NM_008655.1	129
P27	F: TTGTAGAGTAAGTGAAATGG<br>R: CAAGTTTTTCTCATACACAG	NM_009875.4	73
Jun	F: CCCAGTGTTTGTAAATAAGA<br>R: GGGCTACTTTTCAATAGTTT	NM_010591.2	140
Fos	F: CATAGCACTAACTAATCTGT<br>R: CATGATCAGTAACATGACAA	NM_010234.3	241
Bcl-2	F: ATGCCTTTGTGGAACTATATG<br>R: CTACTGCTTTAGTGAACC	NM_009741.5	186
Bax	F: CTCCTCTCCTACTTCGG<br>R: CCCTCCCAATAATTACAAAA	NM_007527.4	210
IL-6	F: GGAAATTTGCCTATTGAAAA<br>R: TGACTCCAGCTTATCTCTTG	NM_001314054.1	165
Cxcl10	F: ATTTAAGTTTGTAAGGACGA<br>R: GATACATACTTGATGACACA	NM_021274.2	184
Mmp13	F: AACTTTCCAAAGAGAGATTT<br>R: CTCAATATCTTCTACCAGTG	NM_008607.2	202
GAPDH	F: CTTAGGTTCATCAGGTAAACTC<br>R: AACATGTAGACCATGTAGTTG	NM_001289726.2	186
